# Verum versus sham tDCS in the treatment of stroke-induced apraxia: study protocol of the randomized controlled trial RAdiCS -“Rehabilitating (stroke-induced) Apraxia with direct Current Stimulation”

**DOI:** 10.1186/s42466-020-0052-y

**Published:** 2020-03-04

**Authors:** Nina N. Kleineberg, Monika K. Richter, Ingrid Becker, Peter H. Weiss, Gereon R. Fink

**Affiliations:** 1 Department of Neurology, Faculty of Medicine and University Hospital Cologne, University of Cologne, Cologne, Germany; 2 Cognitive Neuroscience, Institute of Neuroscience and Medicine (INM-3), Research Center Jülich, Jülich, Germany; 3 Institute of Medical Statistics and Computational Biology, Faculty of Medicine and University Hospital Cologne, University of Cologne, Cologne, Germany

**Keywords:** Anodal transcranial direct current stimulation (tDCS), (Limb) apraxia, Randomized controlled trial (RCT), Two-arm parallel intervention, Stroke, Rehabilitation, Activities of daily living, Cologne Apraxia Screening (KAS)

## Abstract

**Introduction:**

Stroke is the leading cause of acquired disability in western societies. (Motor) cognitive deficits like apraxia significantly contribute to disability after stroke, harming activities of daily living and rehabilitation outcome. To date, efficient therapeutic options for apraxia remain sparse. Thus, randomized controlled trials (RCTs) are warranted.

**Methods:**

Based on promising results of a pilot study, the on-going RAdiCS (**R**ehabilitating stroke-induced **A**praxia with **di**rect **C**urrent **S**timulation) study is a randomized controlled trial, which follows a double-blinded (investigator and patient), two-arm parallel interventional model. It is designed to include 110 apraxic patients (as diagnosed by the Cologne Apraxia Screening, KAS) in the subacute phase after a left hemisphere (LH) stroke. The University of Cologne initiated the trial, which is conducted in two German Neurorehabilitation Centers.

The study aims to evaluate the effect of anodal (versus sham) transcranial direct current stimulation (tDCS) applied over the left posterior parietal cortex (PPC) with an intensity of 2 mA for 10 min on five consecutive days on apraxic deficits. In addition to anodal or sham tDCS, all LH stroke patients undergo a motor (cognitive) training that is performed before and after the stimulation (off-line stimulation).

The primary outcome measure is the (differential) change in the overall KAS score after five daily sessions of anodal versus sham tDCS when compared to the baseline assessment before tDCS. Secondary study outcomes include further apraxia scores, aphasia severity, and measures of motor performance and disability after stroke. All outcome measures are obtained in the post-stimulation assessment as well as during follow-up (3–4 months after tDCS).

**Perspective:**

The RCT RAdiCS shall evaluate in a large number of LH stroke patients whether anodal tDCS (compared to sham tDCS) expedites the rehabilitation of apraxia – over and above additional motor (cognitive) training and standard care. A positive study outcome would provide a new strategy for the treatment of apraxia, which hopefully ameliorates the negative impact of apraxia on daily living and long-term outcome.

**Trial registration:**

Clinical Trials Gov: NCT03185234, registered 14 June 2017 ; Deutsches Register für Klinische Studien: DRKS00012292, registered 01 June 2017.

**Trial status:**

Participant enrollment began on 22 June 2017. The trial is expected to be completed on 30 June 2022.

## Introduction

Stroke is the leading cause of acquired disability in Western societies. Besides primary sensorimotor impairments, cognitive deficits significantly contribute to stroke-related disability. One of those stroke-related (motor) cognitive deficits is apraxia, the “inability to perform specific and predefined actions or to carry out learned and purposeful movements, independently of sensory, motor and (other) cognitive deficits” [14]. Apraxia mainly occurs after a left hemisphere (LH) stroke, with prevalence rates ranging from 30 to 50% [8].

Apraxia may affect different body parts: While limb apraxia involves the hand and the arm, bucco-facial apraxia affects movements and gestures of the face. Apraxia can impair three different motor domains: (i) imitation of (meaningless/meaningful) gestures, (ii) pantomiming the use of objects/tools, and (iii) actual tool use [14]. Notably, apraxic impairments affect both (the contralesional and the ipsilesional) hands.

Apraxia negatively impacts activities of daily living (ADLs [16];). Moreover, apraxic stroke patients show an increased dependency on a caregiver’s assistance and return less frequently to work than patients without apraxia [11]. Thus, apraxia is a poor prognostic factor for the rehabilitation outcome after stroke, calling for the development of novel, efficient therapies. To date, specific therapeutic options for apraxia remain sparse [8]. So far, only two (behavioral) treatment strategies for apraxia have been investigated by randomized controlled trials: gesture training (*n* = 33 [16];) and strategy training (*n* = 113 [7];). Moreover, the evidence for the effectiveness of specific therapeutic strategies for apraxia after stroke is deficient [20].

Recently, the potential of non-invasive brain stimulation, such as transcranial direct current stimulation (tDCS) for the rehabilitation of specific stroke-induced deficits has been recognized (for an overview see [13]). TDCS is a well-tolerated and safe method [2, 4], which modulates neuroplasticity in the human brain by the application of weak currents inducing local effects and effects on connected remote areas [15].

In post-stroke rehabilitation, tDCS was shown to enhance motor and language functions [9] or to ameliorate symptoms of visuospatial neglect [17]. However, up to date, due to a lack of (large) randomized controlled trials (RCTs), there is no Level A recommendation (definitive efficacy) for tDCS in post-stroke rehabilitation for any specific indication [13]. For apraxia, studies in healthy subjects [18], apraxic stroke patients [1, 5], and patients with cortico-basal syndrome [3] showed an improvement in (hand) gesture processing and imitation by anodal tDCS over the left posterior parietal cortex (PPC).

## Methods

### Aim of the trial

A randomized controlled trial (RCT) on the utility of tDCS in apraxia is still missing. Thus, the RCT RAdiCS (Rehabilitating stroke-induced Apraxia with direct Current Stimulation) aims to investigate whether (in addition to motor (cognitive) training) repetitive anodal tDCS applied above the left, ipsilesional PPC can facilitate the recovery of the motor cognitive deficit apraxia during neurorehabilitation in the subacute to chronic phase after LH stroke.

### Study description and study design

This multicenter study is an investigator-initiated, prospective randomized controlled trial (RCT) and follows a double-blinded (patient and investigator) study design with a parallel two-arm interventional model. As an intervention, we apply tDCS with CE-certified DC-Stimulators (NeuroConn, Ilmenau, Germany). In the context of motor rehabilitation after stroke, the clinical trial adheres to the German medical device law (Medizin-Produkte Gesetz, MPG; §23b).

We assume that the verum stimulation (anodal tDCS over the left parietal lobe) leads to a more significant improvement in apraxia severity than sham stimulation [1]. The primary endpoint is the difference in the total score of the Cologne Apraxia Screening (KAS [19]) between the post-interventional investigation and baseline. Thus, the null and alternative hypotheses are:

- **H**_**0**_**:** The change of the motor-cognitive apraxic deficits (as assessed by the KAS) between the baseline assessment and the post-interventional assessment is the same in both treatment groups (verum tDCS and sham tDCS).
- **H**_**1**_**:** The change of the motor-cognitive apraxic deficits (as assessed by the KAS) between the baseline assessment and the post-interventional assessment is different in the verum tDCS group than in the sham tDCS group.

A pilot study following the identical interventional and training procedure was performed before this RCT [1]. Here, 20 apraxic patients showed a standard deviation (SD) of about 7 points in the comparison of the KAS-score between baseline and post-interventional assessment. The difference between the sham- and the verum-interventional group was 4.6 points. Based upon these data, a sample size of 50 per study arm is required to ensure a difference in effect of 4 points at an SD of 7 in a two-sample t-test at a level of significance of 0.05 and a power of 0.8. We added 10% for eventual non-parametric testing, leading to 55 patients per study arm or a total of 110 patients in the intention-to-treat (ITT) collective. Additional participants replace drop-outs that do not fulfill the ITT-criteria (i.e., drop-out before first stimulation).

The primary collective for the analyses is the ITT collective, including all patients with the baseline KAS-score and the application of at least one stimulation. The secondary per-protocol (PP) collective includes all patients with a baseline and post-interventional KAS-score, plus the application of the verum or sham intervention according to the protocol, with the possibility of missing one stimulation, i.e., at least 4/5 stimulations must have been completed. The descriptive analysis will be conducted for the entire study cohort and both study groups. Two-sided statistical tests will be performed with the level of significance set to 0.05. The analysis of effectiveness will primarily be carried out in the ITT population “as randomized”. The PP population (at least 4/5 stimulations received) will be assessed for the sensitivity analysis. The analysis of safety will be conducted in the population “as treated” (at least 1/5 stimulations received).

Including screening, nine study visits are scheduled, eight of which are handled during the inpatient treatment in the neurological rehabilitation centers. The final, ninth (follow-up) visit can also take place at the patient’s home.

**Fig. 1:**
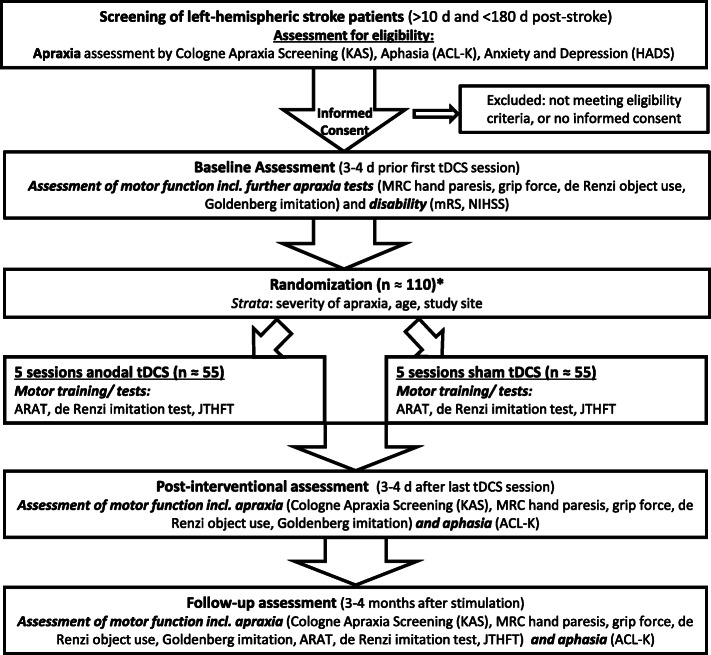
RAdiCS study flowchart. *will be escalated for drop-outs who did not receive a stimulation. Abbreviations: KAS=Kölner Apraxie Screening (Cologne Apraxia Screening), ACL-K=Short version of the Aphasia Check List, HADS=Hospital Anxiety and Depression Scale, MRC = Medical Research Council, mRS = modified Rankin Scale, NIHSS=National Institutes of Health Stroke Scale, ARAT = Action Research Arm Test, JTHFT = Jebsen Taylor Hand Function Test. For a list of the applied tests at a given time point, please, see Table 1

The primary outcome measures are collected at three time points: (1) baseline, i.e., in the week before stimulation, (2) post-intervention, i.e., in the week after stimulation, (3) follow-up, i.e., about three to four months after the stimulation. If patients assent, the neuropsychological and motor tests are recorded on video and re-evaluated for interrater-reliability. Please see the study flow chart Fig. 1 and Table 1 for the time points of the scheduled study visits and the respective motor and neuropsychological tests.

**Table 1:** List of applied tests at a given time point (T0-T8)

			Stimulation						
			S1	S2	S3	S4	S5		
Time point	T0	T1	T2	T3	T4	T5	T6	T7	T8
Method									
Edinburgh Handedness Inventory (EHI)	X								
Hospital Anxiety and Depression Scale (HADS)	X								
Aphasia Check List - Short version (ACL-K)	X							X	X
Cologne Apraxia Screening (KAS)	X							X	X
National Institutes of Health Stroke Scale (NIHSS)		X							
Goldenberg hand- and finger-imitation test		X						X	X
De Renzi test of actual object-use		X						X	X
De Renzi imitation test			XX	XX	XX	XX	XX		X
Action Research Arm Test (ARAT)			XX	XX	XX	XX	XX		X
Jebsen Taylor Hand Function Test (JTHFT)		4X	XX	XX	XX	XX	XX		4X
Modified Rankin Scale (mRS)		X						X	X
Medical Research Council (MRC)		X						X	X
Vigorimeter (for grip force)		X						X	X

T0 = Screening, T1 = Baseline assessment, T2-T6 = assessments at the stimulation sessions (S1-S5), T7 = Post-interventional assessment, T8 = Follow-up assessment

X = single application of the test, XX = application before and after the stimulation (verum or sham tDCS), 4X = 3 times of training and measurement of the 4th trial

### Arms and interventions

After inclusion in the study, participants are randomized in the proportion 1:1 to both study arms, following a stratification by KAS-Score (i.e., apraxia severity; < 68 / ≥ 68 points), age (< 65 / ≥65 years), and site of investigation (Rehabilitationszentrum Godeshoehe e.V., Bonn / MediClin Fachklinik Rhein/Ruhr, Essen).

In the experimental group, anodal tDCS at an intensity of 2 mA is applied for 10 min on five consecutive days. The anodal rubber electrode (size: 5x7cm, 35cm^2^) is placed over the left PPC at position P3 of the 10/20 EEG system, the reference electrode (size: 5x10cm, 50cm^2^) is located supraorbitally on the right forehead. Electrodes are inserted into sponges and dampened with a saline solution to optimize electrode-skin-interface. To fix the electrodes, a rubber band is placed around the head in addition to the EEG cap.

**Fig. 2:**
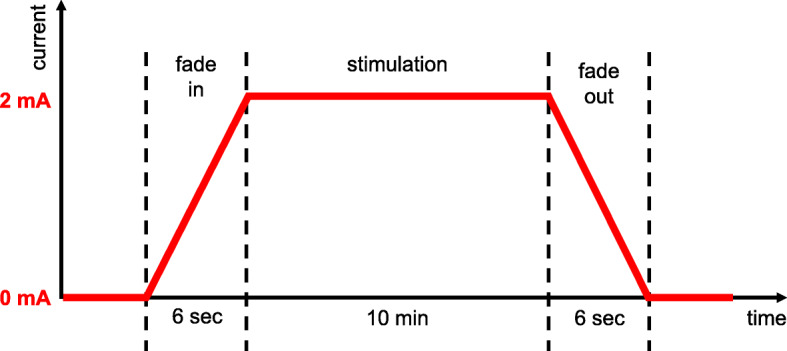
Current flow in anodal tDCS (verum stimulation). After a fade-in phase of 6 s, anodal tDCS is applied at an intensity of 2 mA for 10 min, ending in a fade-out phase of again 6 s

To avoid sudden current changes, a fade-in and fade-out phase is applied, in which the current increases/decreases. Following the recommendation of the NeuroConn company, we ensured that the change of current is less than 0.5 mA per second. Therefore, we used fade-in and fade-out periods of 6 s duration each, resulting in a change of current of 0.5 mA/1.5 s (= 0.333 mA/s, see Fig. 2). During the stimulation, the patient is instructed to sit relaxed in a chair with eyes either closed or opened, without falling asleep (offline-stimulation).

In the control group, a sham stimulation is applied, while the set-up is identical to the anodal tDCS group. The sham stimulation comprises identical fade-in and fade-out-periods (as anodal tDCS) with a short stimulation period of 20s in between, in which a current of 2 mA is applied. This procedure renders the blinding more effective. For the remaining “stimulation” time of 10 min, the tDCS-device performs impedance checks to verify proper electrode functioning and to ensure blinding of the investigator. The mean current applied over the sham stimulation period is below 2 μA and thus cannot modulate cortical excitability. The motor (cognitive) training is performed before and after the stimulation on five consecutive days in both tDCS groups.

The NeuroConn DC-stimulators have a study mode incorporated, which is essential for the double-blind procedure. Two-hundred five-digit codes are pre-programmed, which are either assigned to the verum or sham stimulation. The investigator is blinded to the link between the codes and the stimulation mode. In the process of randomization (see below), each participant is assigned to one of these codes, determining the intervention type (verum/sham). When the code is entered for tDCS application, the display remains identical in both modes during the entire stimulation. The fading of the current at the beginning of the sham stimulation was previously shown to be effective, as patients could not differentiate between verum and sham stimulation [10].

The allocation of codes is conducted via the online randomization service TENALEA (FormsVisions BV, Abcoude, the Netherlands), programmed by the Institute of Medical Statistics and Computational Biology of the University Hospital Cologne. Patients, investigators, and all other involved staff remain blinded for the entire duration of the clinical trial. In the rehabilitation centers, sealed envelopes are deposited for emergency unblinding, enabling the allocation of study arm for each stimulation code.

### Outcome measures

The primary outcome measure is the change of the total score of the Cologne Apraxia Screening (KAS [19];) achieved post-interventionally, compared to the patient’s baseline performance. The KAS is a reliable and valid screening tool [8], assessing bucco-facial and limb apraxia with two types of tasks (pantomime of tool use and imitation). The primary analysis is the comparison of the difference in the KAS-score changes between the two study arms (verum vs. sham stimulation) with a t-test in the ITT collective. If necessary, equivalent non-parametric methods are applied. The differences in KAS-score changes between follow-up and post-interventional assessments, as well as between follow-up and baseline assessments, are compared to evaluate a long-term effect. An analysis of variance (ANOVA) with the factor treatment and the co-factors age, KAS-score classification at baseline, and study site will investigate potential influences of the randomization strata on the treatment effect.

Secondary outcome measures are the changes in the additional neuropsychological and motor tests. Further apraxia assessments include the De Renzi test of actual object-use, the De Renzi imitation test, as well as the Goldenberg hand- and finger-imitation test. Moreover, aphasia is assessed by the short version of the Aphasia Check List (ACL-K). For general motor performance, the force of hand extension is assessed bimanually with the Medical Research Council (MRC) paresis scale, and the grip force is measured with a Vigorimeter (Martin Vigorimeter, medium-sized ball). Furthermore, the Action Research Arm Test (ARAT) is applied to both hands, and the Jebsen Taylor Hand Function Test (JTHFT) is applied to the ipsilesional (left) hand. To document the overall disability after stroke, we use the modified Ranking Scale (mRS).

As (cognitive) motor training, the ARAT, the De Renzi Imitation Test, and JTHFT are conducted before and after the stimulation sessions on the five interventional study visits (S1-S5 = T2-T6).

Additionally, the National Institutes of Health Stroke Scale (NIHSS), the Edinburgh Handedness Inventory (EHI), the Hospital Anxiety and Depression Scale (HADS), and the educational background are assessed at study inclusion.

Secondary outcome measures are assessed at the same time points as the primary outcome measures, i.e., at baseline, post-intervention, and follow-up, and are analyzed analogously to the primary outcome measures (see above).

To evaluate safety and tolerability of the stimulation, all participants receive a questionnaire after the five tDCS sessions about the occurrence of side / adverse effects of stimulation, their severity (1 = absent to 4 = severe), and the association of a putative adverse reaction with the test product (1 = none to 5 = definite). The questionnaire is based on a recommendation by Brunoni and colleagues to systematically assess the safety of tDCS [6]. For all (serious) adverse events ([S]AE), group comparisons are performed with Chi-square (or Fisher-exact) tests for the absolute occurrence as well as severity or causality. Further, to evaluate blinding efficacy, we ask the patients to ‘guess’ whether they received the ‘verum’ or the ‘sham’ stimulation (after all five stimulations have been performed). Further, patients are asked to provide a confidence rating for their guess regarding verum/sham tDCS.

### Eligibility criteria

The criteria for inclusion in the study are defined as follows: (i) age between 18 and 90 years, (ii) LH ischemic stroke in the subacute/ chronic phase (here: > 10 days and < 180 days post-stroke), (iii) clinical presentation of apraxia in the Cologne Apraxia Screening (KAS; cut-off ≤76/ 80 points), and (iv) written informed consent by the patient or the legal guardian.

The criteria for exclusion of the study are defined as follows: (a) inability of the stroke patient to provide informed consent; in case of a legal guardian: the legal guardian is not available or declines consent; (b) pregnancy and breastfeeding; (c) patients with clinically manifest stroke before the index stroke; (d) malignant disease with affection of the central nervous system; (e) estimated life expectancy < 12 months; (f) current alcohol or drug addiction or other addictive disease (exception: nicotine); (g) current clinically manifest psychiatric disorders, such as schizophrenia or severe depressive episode; (h) epileptic seizure within the past two years; (i) intake of anti-epileptic drugs for prophylaxis of epileptic seizures; (j) continuous medication during the interventional phase with benzodiazepine or antipsychotics of high potential; (k) enrollment in other studies with brain stimulation in the period after the index stroke; (l) heart pacemaker; (m) electrodes for deep brain stimulation or other metal implants in the head; (n) craniectomy or cranial trepanation; (o) vulnerable skin lesions at planned electrode positions; and (p) poor motivation or cooperation.

In this study, LH stroke patients with apraxia are recruited during their inpatient stay in rehabilitation centers. The index stroke is diagnosed according to clinical standards (preferably including neuro-imaging with either cerebral computed tomography (CT) or cerebral magnetic resonance imaging (MRI)). If the patient provides written consent, the corresponding brain scans are obtained from the primary hospital and will be used for lesion mapping in the final reporting.

Patients with LH stroke are screened by the investigator (medical doctor). For the screening procedure, written informed consent is obtained. After that, inclusion and exclusion criteria are checked, and the KAS, the ACL-K, the EHI, and the HADS are applied. In case that all eligibility criteria are met, the patient is informed about the study procedures in detail, the interventional product, including potential side effects, and the randomization procedure. The study informed consent form, including the insurance documents, is handed to the patient. Then, time for consideration of at least 24 h is given until study inclusion takes place. In the case of a legal guardian, the procedure is carried out with the guardian and patient together.

### Contacts (sponsors and collaborators, investigators)

The study was initiated by the Department of Neurology of the University Hospital of Cologne (Principal Investigator: Gereon R. Fink) and is conducted in two neurological rehabilitation centers in Germany: the Rehabilitationszentrum Godeshoehe e.V. in Bonn and the MediClin Fachklinik Rhein/Ruhr in Essen.

The monitoring is carried out by the Contract Research Organization (CRO) Cato Europe GmbH, Cologne. To ensure adherence to the rules of good clinical practice (GCP; e.g., compliance with the study protocol, the quality of the documentation, including regular verification of the electronic case report forms of each study participant), a monitor regularly inspects the study centers.

The study was approved on 23 May 2017 by the Ethics Committee of the University Hospital of Cologne and follows the guidelines of the declaration of Helsinki and the DIN EN ISO 14155:2011.

### Perspective

The RAdiCS study aims to investigate whether compared to a sham stimulation non-invasive brain stimulation, here: anodal tDCS applied over left parietal cortex, has an additional effect, i.e., over and above a motor (cognitive) training, on the rehabilitation of apraxia in patients with LH stroke during their in-patient stay in a rehabilitation center. Besides the fact that apraxia has a negative impact on activities of daily living and the outcome after stroke, the clinical relevance of this trial is further substantiated as we examine patients suffering from persistent apraxia after the acute phase (i.e., > 10 days post-stroke; cf. [12]). The study visits were carefully designed to fit into the daily routine rehabilitation treatment to assure overall feasibility and avoid any overburdening of the patients. All study visits are performed in addition to the regular rehabilitation regimen. Thus, anodal tDCS constitutes an add-on therapeutic option. A positive outcome of the study would provide a new effective strategy for the treatment of apraxia, which is needed.

## Data Availability

The datasets used and/or analyzed during the RCT will be available after completion of the study from the corresponding author on reasonable request.

## Abbreviations

ACL-K: Short version of the Aphasia Check List
ARAT: Action Research Arm Test
HADS: Hospital Anxiety and Depression Scale
JTHFT: Jebsen Taylor Hand Function Test
KAS: Kölner Apraxie Screening (Cologne Apraxia Screening)
LH: Left hemisphere
MRC: Medical Research Council
mRS: Modified Rankin Scale
NIHSS: National Institute of Health Stroke Scale
RCT: Randomized controlled trial
tDCS: Transcranial direct current stimulation
